# Big Data Opportunities for Global Infectious Disease Surveillance

**DOI:** 10.1371/journal.pmed.1001413

**Published:** 2013-04-02

**Authors:** Simon I. Hay, Dylan B. George, Catherine L. Moyes, John S. Brownstein

**Affiliations:** 1 Spatial Ecology and Epidemiology Group, Department of Zoology, University of Oxford, Oxford, United Kingdom; 2 Fogarty International Center, National Institutes of Health, Bethesda, Maryland, United States of America; 3 Department of Pediatrics, Harvard Medical School and Children's Hospital Informatics Program, Boston Children's Hospital, Boston, Massachusetts, United States of America

## Abstract

Simon Hay and colleagues discuss the potential and challenges of producing continually updated infectious disease risk maps using diverse and large volume data sources such as social media.

**Summary Points**

- Systems to provide static spatially continuous maps of infectious disease risk and continually updated reports of infectious disease occurrence exist but to-date the two have never been combined.
- Novel online data sources, such as social media, combined with epidemiologically relevant environmental information are valuable new data sources that can assist the “real-time” updating of spatial maps.
- Advances in machine learning and the use of crowd sourcing open up the possibility of developing a continually updated atlas of infectious diseases.
- Freely available dynamic infectious disease risk maps would be valuable to a wide range of health professionals from policy makers prioritizing limited resources to individual clinicians.

## Where Are the Diseases of Clinical Significance?

It is perhaps surprising to state that we have an extremely poor knowledge of the global distribution of the vast majority of infectious diseases [1]. A review of all infectious diseases of clinical significance has revealed it would be of public health benefit to map about half of these conditions; yet, astonishingly, only 2% (seven of 355) have been mapped comprehensively [2]. This geographical ignorance frustrates a variety of clinical, epidemiological, and public health aspirations.

Here we argue that this information gulf has serious implications for global public health surveillance and that too little attention is given to spatial epidemiology in international preparedness planning. Stated simply, how can we gauge the risk posed by new infectious disease outbreaks if we have only the crudest understanding of their natural geographical range? Additionally, how do we prioritise useful intelligence in the growing deluge of Big Data [3]–[5] if the contemporary geographical distribution of these infectious disease threats is unknown? We suggest that it should be a policy priority to improve the ability to triage spatially, infectious disease outbreak alerts [6],[7].

## How Do We Map Infectious Diseases?

**Figure 1:**
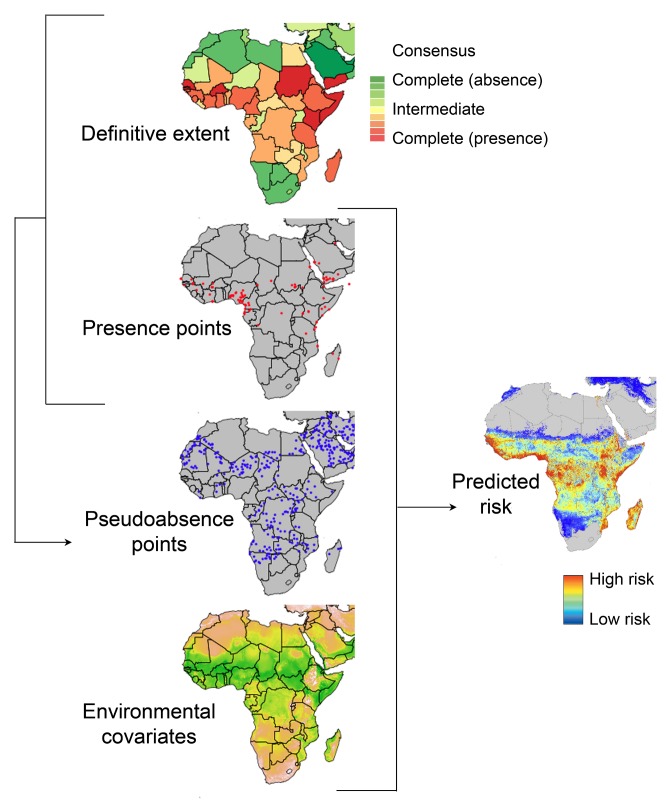
A schematic overview of the process of predicting spatial disease risk. The *definitive extent* of infectious disease occurrence at the national level (red is certain presence, green is certain absence) [16] is combined with assemblies of known occurrence, presence *points* (red dots), to generate putative *pseudo-absence points* (blue dots). The *presence* and *pseudo-absence* data are then used in the analyses, with selected *environmental covariates* to predict *disease risk*, formally the probability of occurrence of the target disease. In this example a risk map of dengue is shown, shaded from low probability of occurrence in blue to high probability of occurrence in red [8]. The arrows represent data flows.

To explore the factors hindering progress, we need to consider how traditional methods are used to map disease. We illustrate this in Figure 1 using a schematic of the cartographic process applied recently to map dengue [8],[9]. The objective is to make a continuous map of the entire geographical range of a disease from a sample of locations where the disease has been observed [10],[11]. In the ecological literature this is described as identifying the fundamental niche of the target organism [12],[13]. In our application it is the fundamental niche of an infectious disease. It is rare for any organism or disease to fully exploit all of the environmental space that is available to it, due to a whole host of evolutionary, biogeographical and ecological factors, so to help guide the mapping process we use evidence-based expert knowledge to demark the crude global limits of a disease—its definitive extent or realised niche.

Figure 1 shows the process used to generate a continuous data layer of disease risk, in this example dengue. The process starts with records of disease occurrence obtained from the literature [14], web reports [3], and GenBank [15] that are used to define the definitive extent of the disease [16] and to populate a database of occurrence points where the disease has been reported. Because it is rare for disease absence to be recorded, a common practice in niche mapping and modelling is to infer absences [17],[18]. The definitive extent and occurrence point data are used to infer plausible pseudo-absence points for further analysis [9].

To complete the process illustrated in Figure 1, a range of epidemiologically relevant environmental covariates are also assembled. These covariates, such as temperature and rainfall, must cover the area over which prediction is desired. Statistical techniques are then used to characterise points of presence and pseudo-absence against the range of covariates assembled [8],[9]. In this instance we favoured the Boosted Regression Tree technique due to favourable comparative reviews of performance, statistical flexibility, and community support evidenced by well documented and freely available R-code [19],[20]. These relationships are then used to predict the probability that the disease occurs at each location and thereby generate a risk map with a quantified measure of uncertainty.

Canonically, the output risk surface is where the mapping process ends, which further compounds the problem of the paucity of infectious disease mapping. This is in part due to the very labour intensive nature of assembling (most often from the peer-reviewed literature, for example, over 2,000 published articles contributed data to the latest map of malaria vectors [21]) and then geo-positioning the required information. Usually, trained analysts do this manually, so capacity for update and refinement is limited by human resources. Moreover, our strong perception of disease maps as static is clearly flawed because disease risk can change rapidly in space and in time and since knowledge about the distribution of diseases now improves daily [3]–[5],[22],[23], risk maps become quickly outdated.

## Can We Use Big Data Approaches to Routinely Map All of These Infectious Diseases?

The process described above provides a continuous risk map in space that is static in time. Conversely HealthMap (www.healthmap.org) provides continually updated disease occurrence points but not continuous spatial data. Can we conceive of spatially continuous risk maps being updated in “real-time”—as frequently as new occurrence data are assimilated? The conceptual bridge of imagining spatial modelling as a continuous process in time is achieved simply by linking the output risk map back to the data inputs to create a feedback loop. This is important as it facilitates the novel step of spatial triage of new occurrence information (see below) and critically, the potential for multiple iterations of the map with continuous improvement by adding a machine learning element. This conceptual shift towards evolving maps, in combination with the increased availability of novel digital data sources [5],[22], is now dissected in the context of “Big Data.”

Big Data is a term used to describe information assemblages that make conventional data, or database, processing problematic due to any combination of their size (volume), frequency of update (velocity), or diversity (variety) [24]. These “volume, velocity, and variety” descriptors have proven useful themes with which to explore opportunities and challenges of Big Data [24] and are emulated here. Each part of this mapping process can be radically improved with a Big Data approach, and the extent of the Big Data challenge is highlighted in Table 1. These challenges are discussed in turn.

**Table 1:** An assessment of the challenges of using Big Data in disease mapping.

	Definitive Extent	Occurrence Point	Pseudo-Absence Point	Environmental Covariates	Risk Prediction
Volume (scale)	+++	+++	+	+++	+++
Velocity (frequency)	+++	+++	++	+++	+++
Variety (diversity)	++	++	+	+	+

The potential Big Data challenges in each stage of an iterative mapping process are highlighted in the table. The columns represent each of the mapping stages defined in Figure 1. The rows reflect the volume, velocity, and variety descriptors of data contributions. The future Big Data challenge in relation to infectious disease risk mapping is as follows: low (+), medium (++), and high (+++).

It is well established that a huge amount of novel data are being generated that will make important contributions to temporal public health surveillance [5],[22]. The secondary use of passive search query and micro-blogging data as well as actively collected crowd-sourced data for disease surveillance has been well documented and validated for major public health events, including influenza and dengue epidemics [22],[23],[25]. Though these data sources demonstrate significant noise and require continual model fine tuning, the sheer volume of health outcome related searches and personal accounts presents incredible new opportunities to monitor population health in real time. It is less well appreciated that this information could also be used to build definitive extents and databases on the occurrence of many diseases [2]. The volume, velocity, and variety of occurrence information from these sources will increase rapidly and transform our ability to create geographical baselines for a range of diseases. These novel data sources come with issues of reliability so it is important that the machine learning process is calibrated for known reporting bias and the triage process assigns a weighting to each data point as a measure of reliability. This weighting is an integral part of the niche mapping techniques used and feeds into the measure of uncertainty output for each location. An increasing proportion of these new data are geo-positioned at source. Moreover, machine learning approaches to automate geo-positioning of disease reports [26], especially when combined with human oversight and crowdsourcing (outsourcing tasks online to volunteers) [27],[28], can further radically lower the logistical barriers to positioning this information.

In the era of satellite sensors, a diversity of epidemiologically relevant environmental information can be sourced globally at daily intervals [29]. Big Data volume, velocity, and variety challenges are involved in moving from the traditional processing of synoptic averages of covariates to harnessing a wider variety of temporally rich information that can be matched in time with the new occurrence information. This closer temporal matching of disease outbreaks with covariates may improve the accuracy of mapping models, allowing for the possibility of seasonally tailored geographic baselines and may help improve traditional temporal surveillance by facilitating early warning of epidemiologically relevant environmental changes.

Perhaps the most important development in relation to Big Data is the conceptual move from static to improving and evolving risk maps. Taking further our example of dengue mapping (Figure 1), the first evidence-based risk map generated can be used to help triage the information content of new reports before running the next map iteration. For example, disease reports located nearby existing records and with a high-predicted probability of occurrence are not alarming; we expect the disease to occur here from the history of reporting and the suitability of the environment. Furthermore, such reports will not substantially alter the risk map and are thus of low priority to analysts. Conversely, a disease outbreak far away from observed occurrence is alarming, and more so if it occurs in an area biologically suitable for the disease. It should be investigated and, if verified, contribute to improving next iterations of the map. It is easy to imagine how these automated learning processes, supervised by expert analysts, could be deployed in tandem for all diseases of concern, transforming our spatial intelligence, surveillance, and preparedness.

## The Challenges Ahead

The biggest obstacles to incorporating a continuous spatial mapping component to routine biosurveillance will be demonstrating the feasibility and sustainability of this undertaking and engaging the audience. We have focussed here on applications for biosurveillance but it is important to emphasise the wider audiences. First, one should never underestimate the value of risk maps in helping to illustrate the extent of a public health problem [30]. Second, addressing the paucity of spatial information on infectious disease distributions will transform our understanding of their environmental determinants and help radically improve our understanding of the factors that promote disease diversity [31] and emergence [32]. Third, a comprehensive atlas of contemporary distributions would be of considerable benefit to improve future assessments of the burden of disease [33]. The audience for risk maps that are continuous in time and space includes agencies who need to prioritise limited resources and respond to changing disease patterns, public and private R&D pipelines who need to assess value and plan research strategy, logistics groups who need to optimise the roll out of new interventions/treatments, and clinicians who want to accurately diagnose infectious diseases in local populations and returning travellers.

We have already argued that this mapping ambition is made tractable by automating many of the laborious steps in primary data acquisition and positioning. The Big Data revolution is already underway and harnessing the useful information in these new data sources will involve collaborations with computer scientists at the forefront of machine learning and with those who have had success in engaging communities [27]. The evidence shows that motivating people to devote some of their “cognitive surplus” to crowd sourcing is possible, so long as the products and benefits are immediately available to all for the common good. We have seen the rise of crowdsourcing influenza surveillance with participatory systems such as Flu Near You in the United States (www.flunearyou.org) and Influenzanet in the EU (www.influenzanet.eu), which now boast nearly 100,000 volunteers combined. From the outset all infectious disease data and derived maps should be made freely available to ensure engagement. This will also facilitate the uptake of new resources and their consideration by policy makers. Once the primary investment in the software platform is complete, and the community established, sustainability increases because demands for user inputs decrease as the software learns and the mapped outputs become increasingly stable. The ultimate vision is to democratise the platform by providing the code to all interested authorities.
